# Structure-based identification of bioactive phytochemicals targeting kallikrein-related peptidase 2 for prostate cancer therapy

**DOI:** 10.3389/fchem.2025.1553987

**Published:** 2025-03-26

**Authors:** Deeba Shamim Jairajpuri, Afzal Hussain, Mohamed F. Alajmi, Taj Mohammad, Anas Shamsi, Md. Imtaiyaz Hassan

**Affiliations:** ^1^ Department of Medical Biochemistry, College of Medicine and Health Sciences, Arabian Gulf University, Manama, Bahrain; ^2^ Department of Pharmacognosy, College of Pharmacy, King Saud University, Riyadh, Saudi Arabia; ^3^ Centre for Interdisciplinary Research in Basic Sciences, Jamia Millia Islamia, New Delhi, India; ^4^ Center of Medical and Bio-Allied Health Sciences Research (CMBHSR), Ajman University, Ajman, United Arab Emirates

**Keywords:** kallikrein-related peptidase 2, prostate cancer, molecular docking, virtual screening, drug discovery, MD simulations

## Abstract

Kallikrein-related peptidase 2 (KLK2) is a serine protease exhibiting antiangiogenic properties through proteolytic activity. KLK2 is overexpressed in prostate cancer and plays a pivotal role in cancer progression, establishing it as a potential therapeutic target. Despite the promising results of small molecule inhibitors targeting KLK2 in prostate cancer treatment, there are still many challenges in the development and application of these inhibitors. As a consequence, very few KLK2 inhibitors have advanced to clinical trials because of issues with specificity and selectivity. Moreover, the precise mechanisms underlying KLK2’s interactions with small molecule inhibitors remain inadequately understood. This study used structure-based virtual screening of a phytochemical library and found three compounds, Phaseolin, Withaphysalin D, and Nicandrenone, as potential KLK2 inhibitors. These compounds exhibited high binding affinities (−8.9 to −8.8 kcal/mol), favorable pharmacokinetic profiles, and stable interactions with KLK2’s catalytic residues (including His65) in docking studies. Their binding was further validated through MM-PBSA free energy calculations, which confirmed energetically favorable interactions with KLK2. The findings suggest that these phytochemicals have a high potential to be exploited as novel KLK2 inhibitors with improved efficacy. While experimental validation of enzymatic inhibition and antitumor efficacy is required, this study provides a structural and mechanistic foundation for advancing these candidates into preclinical testing. These results also highlight the use of phytochemical libraries and dynamics-driven virtual screening in developing targeted therapies for prostate cancer.

## 1 Introduction

Human kallikrein 2, also known as kallikrein-related peptidase 2 (KLK2), is a serine protease with trypsin-like activity predominantly expressed in the prostate, making it a compelling target for prostate cancer therapy (Mohammadi et al., 2024). KLK2 is co-expressed with kallikrein 3 (KLK3), commonly known as prostate-specific antigen (PSA), within the same tissues (Shang et al., 2014). The concentration of KLK2 in seminal plasma is about 1% of PSA. Androgens and androgen receptor (AR) signaling regulate the overall expression of the *KLK2* gene. Hence, KLK2, like PSA, can also be a biomarker for various cancers, especially prostate cancer (Motta et al., 2023). Apart from PSA inhibitors, KLK2 inhibitors have also been developed as small-molecule therapeutics for multiple malignancies, including prostate cancer, (Lövgren et al., 1999). Prostate cancer has emerged as a significant health threat, ranking as the second most diagnosed cancer and the sixth leading cause of tumor-related deaths in men worldwide (Schröder, 2010; Sharma et al., 2024). It predominantly affects middle-aged men between 45 and 60 years of age and represents the highest cause of cancer-related mortality in Western countries. Diagnostic methods for prostate cancer include the PSA test, magnetic resonance imaging (MRI), prostate biopsy, and digital rectal examination (Hannu et al., 2014).

The disease is influenced by several risk factors, including family history, ethnicity, age, obesity, and environmental factors, and it exhibits considerable heterogeneity based on genetic and lifestyle variations (Hoffman, 2011). Prostate cancer remains the second leading cause of cancer-related deaths in men globally, with over 1.4 million new cases annually. Current therapies, including androgen deprivation and chemotherapy, often fail due to drug resistance and systemic toxicity. Targeting KLK2 offers a promising strategy to circumvent these limitations by inhibiting tumor-specific pathways critical for metastasis and angiogenesis. The critical role of KLK2 in tumor development, metastasis, and angiogenesis inhibition, along with its selective expression in prostate tissue, highlights its potential as a therapeutic target in prostate cancer. The identification and evaluation of novel KLK2 inhibitors have garnered substantial pharmacological interest. These inhibitors hold promise for developing targeted therapies for prostate cancer and other malignancies associated with heightened protease activity (Geary and Salem, 2013).

Recent efforts have identified a limited number of KLK2-mediated prostate cancer therapeutics, including the JNJ-78278343 (Janssen Pharmaceuticals) and AC0176 (Zhang and Chadha, 2024). Small molecules such as PPACK (H-D-Phe-Pro-Arg chloromethyl ketone) and Benzamidine have been co-crystallized and evaluated with KLK2, demonstrating promising inhibitory potential (Skala et al., 2014). Preclinical studies of these agents have shown encouraging antitumor activity, though challenges such as off-target effects, variable specificity, and pharmacokinetic limitations persist (Martin et al., 2023). While these candidates represent significant strides in KLK2-targeted therapy, no inhibitors have yet achieved FDA approval. The field remains nascent, with research constrained to a small pipeline of experimental agents, underscoring the need for further mechanistic exploration and clinical validation. Therefore, it is essential to develop safe and selective KLK2 inhibitors for treating prostate cancer and related conditions (Joniau et al., 2012).

Computational drug discovery has brought about a new dimension in discovering novel therapeutic agents because the approach is fast and less expensive compared to experimental high-throughput screening (Naithani and Guleria, 2024). Among the fundamental methods in this field, virtual screening allows the recognition of potential bioactive compounds based on their interaction with target proteins (Naqvi et al., 2018). This method has received significant interest in drug discovery because it can rank compounds for experimental testing. Plant-derived secondary metabolites known as phytochemicals are gaining popularity in treating diseases, especially cancer (Swetha et al., 2022). As phytochemicals exhibit various biological activities, these compounds are an attractive source for new drug leads (Bharate and Lindsley, 2024). Thus, it has become possible to use virtual screening of phytochemicals to identify bioactive compounds with specific targeting capabilities like the ability to inhibit KLK2.

This study employed a structure-guided virtual screening approach to identify potent inhibitors of KLK2. The binding dynamics of the discovered phytochemicals and KLK2 were analyzed using all-atom molecular dynamics (MD) simulations. A total of 11,699 phytochemicals from the IMPPAT 2.0 database were screened. IMPPAT 2.0 is a wide-ranging database curated from over 100 traditional Indian medicine books, more than 7,000 research papers, and additional sources, providing an extensive repository of phytochemical data (Vivek-Ananth et al., 2023). This structure-based screening approach utilized plant-derived compounds from the IMPPAT 2.0 database to evaluate the structural stability of the docked complexes, offering a promising pathway for discovering safe and effective KLK2 inhibitors.

## 2 Material and methods

### 2.1 Computational resources

Bioinformatics tools like InstaDock (Mohammad et al., 2021), Discovery Studio Visualizer (Visualizer, 2005), GROMACS (Van Der Spoel et al., 2005), PyMOL (DeLano, 2002), and others were used for molecular docking, visualization, and simulation studies. For data evaluation and retrieval, numerous resources were used, such as RCSB-Protein Data Bank (PDB) (Berman et al., 2000), SwissADME (Daina et al., 2017a), pkCSM (Pires et al., 2015), Way2drug for PASS analysis (Druzhilovskiy et al., 2017), etc. The three-dimensional structure of the KLK2 protein was downloaded from the RCSB Protein Data Bank (ID: 4NFF). All water molecules, co-crystallized heteroatoms, and ligands were deleted from the original structure. The KLK2 structure was remodeled using PyMod 3.0 (Janson et al., 2017) to enhance its suitability for docking studies. The PDB structure 4NFF was used for docking and molecular simulations. Variations in KLK2 residue numbering across different PDB entries arise due to construct-specific differences, such as truncations or expression tags. To ensure consistency, we aligned the active-site residues in 4NFF with the UniProt sequence (P20151). A library of 11,699 phytochemical compounds, adhering to Lipinski’s rule of five (RO5), was obtained from the IMPPAT 2.0 database and subsequently docked with the remodeled KLK2 structure (Vivek-Ananth et al., 2023).

### 2.2 Molecular docking-based screening

Molecular docking is a common approach in drug discovery for evaluating the binding affinity, selectivity, and specificity of small-molecule candidates toward their target proteins (Muhammed and Aki-Yalcin, 2024). Docking simulations were performed using InstaDock v1.2, a free and efficient platform for virtual screening of potential drug candidates. The blind search grid was configured with dimensions of 50 Å × 53 Å × 51Å, centered at X: 29.432Å, Y: 11.13Å, and Z: 10.963Å, to encompass the KLK2 binding site. This docking grid was large enough to accommodate the entire protein and cover all heavy atoms, ensuring that each ligand could explore the most favorable binding pocket(s). This search space was optimized to provide comprehensive coverage while maintaining computational efficiency, preventing excessive search times without compromising accuracy. Docking results were filtered based on affinity scores to identify suitable conformers for interaction analysis. PyMOL and Discovery Studio Visualizer analyzed the interactions within the KLK2 binding pocket.

### 2.3 ADMET evaluation

SwissADME (Daina et al., 2017b) and pkCSM (Pires et al., 2015) servers were utilized to evaluate the pharmacokinetic properties of the filtered compounds identified as high-affinity binding partners of KLK2 through molecular docking. These tools predict critical physicochemical and pharmacokinetic characteristics of the compounds, adhering to Lipinski’s rules. Additionally, the PAINS filter was applied to exclude compounds that might produce false-positive results in bioassays (Lipinski, 2004). Compounds that exhibited significant ADMET properties and did not display PAINS patterns were selected for further analysis, ensuring a robust pharmacokinetic profile for potential drug candidates.

### 2.4 Biological activity prediction

PASS analysis is a highly valuable tool for assessing chemical compounds’ biological activities and interactions (Filimonov et al., 2014). Following ADMET analysis, PASS was employed further to evaluate the biological potential of the screened phytochemicals. The PASS server provides results in terms of “Probability to be active (Pa)” and “Probability to be inactive (Pi),” where a higher Pa value indicates an increased likelihood of a compound exhibiting the associated biological activity.

### 2.5 Interaction analysis

After PASS and ADMET evaluations, the two-dimensional polar interactions between the compounds and KLK2 were analyzed using PyMOL, recording interactions within a distance of 3.5 Å. Discovery Studio Visualizer confirmed the interactions between the phytochemicals and the KLK2 binding pocket. The numbering in PDB ID: 4NFF differs from UniProt due to sequence offsets, and the structure was renumbered accordingly (UniProt ID: P20151) to maintain consistency with the reference annotation. Only compounds interacting with critical residues within the KLK2 binding site and demonstrating strong binding were selected for further analysis. The binding site and docking positions of KLK2 (PDB ID: 4NFF) were used as references to validate the docking outcomes. Multiple binding conformations of ligands were generated using InstaDock, and the most effective conformation for each ligand was identified through docking and interaction analysis. Additionally, the three-dimensional binding patterns of each ligand with KLK2 were visualized using PyMOL, providing insights into their binding modes.

### 2.6 MD simulations

The application of MD simulations has significantly advanced drug development in recent years (Hassan et al., 2023). MD simulations provide detailed insights into the activity of biomolecules, such as proteins, with high temporal resolution and atomic precision (Hollingsworth and Dror, 2018). In this study, the selected phytochemicals were subjected to MD simulation studies using the GROMACS suite for 300 ns following docking and evaluation steps. At a constant temperature of 300K, the Chemistry at Harvard Macromolecular Mechanics (CHARMM) force field was used in the simulation. The CGenFF (Vanommeslaeghe and MacKerell, 2012) webserver produced the force field parameters and ligand topology for each small molecule. Protein-ligand complexes were solvated with the TIP3P water model in a 10 Å cubic box of water using the gmx solvate module. Energy minimization was performed using the steepest descent algorithm, followed by charge neutralization. Each system underwent a 1,000 ps equilibration phase at constant volume, during which the temperature was gradually increased from 0 to 300 K under periodic boundary conditions. Quality control metrics such as density, enthalpy, kinetic energy, and volume were monitored to ensure simulation accuracy. Trajectories were evaluated for various conformational parameters further to analyze the dynamics of KLK2 and its ligand complexes. Graphs and figures depicting KLK2 interactions and stability were generated using QtGrace (Turner, 2005).

### 2.7 Essential dynamics

Essential dynamics aims to find large-scale motions in biomolecules, and it targets the motions that govern the dynamics of the system (Papaleo et al., 2009). Principal component analysis (PCA) simplifies the data and retains essential variations, best illustrating the primary conformational changes in protein-ligand interactions. This approach helps reduce the data and makes it easier to visualize the various dynamics. This is supported by free energy landscape (FEL) analysis, which gives the energy map of the system the most stable conformations. In combination with PCA and FEL, the present study provides a clear picture of the stability and flexibility of the protein-ligand complexes. It will facilitate the design of particular therapeutic interventions.

### 2.8 MM/PBSA calculations

Molecular Mechanics/Poisson-Boltzmann Surface Area (MM/PBSA) is a widely applied method for estimating the binding free energy between a protein and a ligand (Genheden and Ryde, 2015). It combines molecular mechanics calculations with solvation energy estimations to provide insights into the stability and affinity of molecular interactions. For this study, MM/PBSA calculations were performed to evaluate the binding affinities of the ligand-bound KLK2 complexes. A 10 ns segment was extracted from the stable region of each MD simulation to ensure an accurate representation of the binding interactions. Binding free energy components were computed using the gmx_mmpbsa package, which applies the MM/PBSA approach based on the following equation:

$$\Delta G_{\text{Binding}}=G_{\text{Complex}}\;‐\;\left(G_{\text{Protein}}+G_{\text{Ligand}}\right)$$

where *G*_Complex_ signifies the total free energy of the binding complex, and *G*_Protein_ and *G*_Ligand_ are the measure of total free energies of KLK2 and the bound ligands, respectively.

## 3 Result and discussion

### 3.1 Molecular docking screening

A library of 11,699 phytochemicals from the IMPPAT 2.0 database was utilized to conduct a structure-guided virtual screening strategy to identify potential KLK2 inhibitors. After the docking process, the docking software generated log and output files for each compound, including affinity scores and docked poses. This step facilitated the elimination of phytochemicals based on unsuitable binding affinities and binding poses. The screening process identified many promising hits with high binding affinity towards the KLK2 binding cavity, making them viable candidates for further evaluation as potential KLK2 inhibitors. Following a detailed analysis of the docked output, 15 hits were selected from the initial 11,699 compounds based on their significant binding affinities (computational Δ*G* scores), which ranged from −9.6 to −8.7 kcal/mol (Table 1).

**TABLE 1:** Top 15 phytochemicals with their binding affinity with KLK2. Ligand efficiency values are in kcal/mol/non-H atom.

S. No.	IMPPAT ID	Phytochemical	2D structure	Binding affinity (Kcal/mol)	Ligand efficiency
1	IMPHY009090	Ugonin A	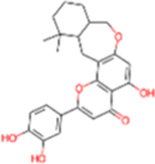	−9.6	0.3097
2	IMPHY007679	Bismurrayaquinone A	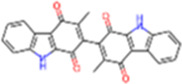	−9.2	0.2875
3	IMPHY012556	Argentine	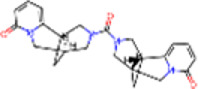	−9.1	0.3033
4	IMPHY002553	Parvisoflavone B	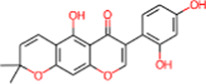	−9.1	0.35
5	IMPHY003078	Kuwanon D	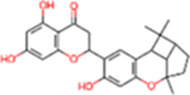	−9.1	0.2935
6	IMPHY014146	Bianthraquinone	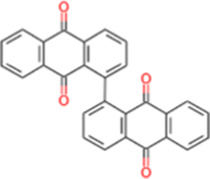	−9	0.2812
7	IMPHY007730	Californine	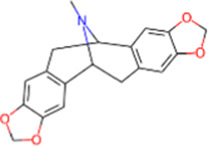	−8.9	0.3708
8	IMPHY008900	Withaphysalin D	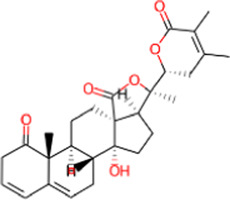	−8.9	0.2618
9	IMPHY010989	Nicandrenone	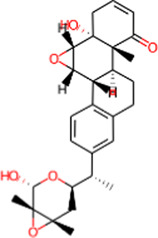	−8.9	0.2618
10	IMPHY000058	Ovalichromene B	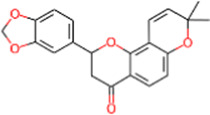	−8.8	0.3385
11	IMPHY007336	Phaseolin	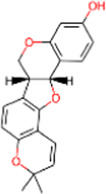	−8.8	0.3667
12	IMPHY002700	Withaphysalin A	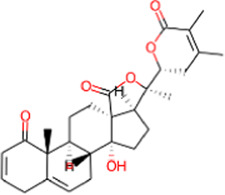	−8.8	0.2588
13	IMPHY009050	Picrasidine T	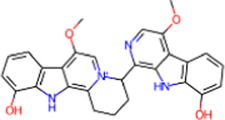	−8.8	0.2444
14	IMPHY003705	(−)-Ephedradine A	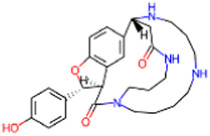	−8.7	0.2417
15	IMPHY003706	Aphelandrine	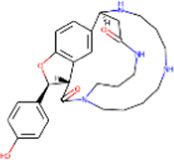	−8.7	0.2417
16	IMPHY017760	Valeronitrile	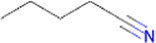	−2.9	0.4833
17	PPACK (PDB ID: OG6)	H-D-Phe-Pro-Arg chloromethyl ketone	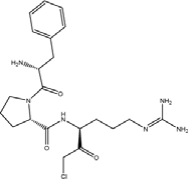	−6.3	0.21
18	Benzamidine (PDB ID: BEN)	Benzamidine	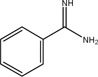	−5.8	0.6444

To benchmark these values, docking was performed under identical conditions for known KLK2 inhibitors PPACK and Benzamidine, which showed lower binding affinities of −6.3 kcal/mol and −5.8 kcal/mol, respectively. To assess ligand specificity, negative control, Valeronitrile, with weak binding affinity (−2.8 kcal/mol) was included in the docking study. Unlike the top-ranked inhibitors, this compound exhibited poor interactions with KLK2’s active site, confirming the specificity of the identified phytochemicals. Since more negative binding energy values (e.g., Δ*G*) indicate a more stable receptor-ligand complex, as seen in molecular docking studies, these compounds are anticipated to form highly stable complexes with KLK2. While this suggests that our phytochemicals may form more stable interactions with KLK2, it is essential to recognize that PPACK and Benzamidine have been experimentally validated. Therefore, further *in vitro* validation is required to confirm whether the computationally predicted higher binding affinity translates into stronger inhibitory activity in biological assays.

### 3.2 Physicochemical and pharmacokinetic properties

In computer-aided drug discovery, predicting ADMET properties is critical in screening compounds. To assess the ADMET profiles of the top fifteen hit compounds, we employed the pkCSM online server. SMILES representations of the compounds, obtained using Discovery Studio Visualizer, were used as input for ADMET prediction (Jain et al., 2022). Evaluating a compound’s physicochemical and pharmacokinetic characteristics is essential to determine its potential as a drug candidate and its likelihood of clinical success (Lagorce et al., 2017). The ADMET properties and PAINS filter assessments were conducted using pkCSM and SwissADME tools (Parmar et al., 2022). These evaluations focused on the top 15 compounds with the highest binding affinity towards the KLK2 active site. Initially prioritized based on their docking scores, the compounds underwent ADMET analysis to identify the most promising hits (Bhakhar et al., 2021). Here, the screened compounds were eliminated based on parameters such as PAINS alerts, hepatotoxicity, AMES toxicity, solubility, and carcinogenicity. The ADMET results highlight Phaseolin, Withaphysalin D, and Nicandrenone as the most viable inhibitor compounds with appropriate pharmacokinetic properties (Table 2). The selection of these compounds was based on multiple parameters beyond binding affinity. Compounds with PAINS alerts, hepatotoxicity, AMES toxicity, poor solubility, or carcinogenicity were excluded. These compounds demonstrated favorable pharmacokinetic profiles and low toxicity predictions, supporting their potential for safe systemic administration. Furthermore, their properties indicate suitability for development as oral therapeutics for prostate cancer treatment.

**TABLE 2:** Pharmacokinetic properties of the screened phytochemicals showing different properties of ADMET parameters. BBB, blood-brain barrier; PPB, plasma protein binding, Proper Value: therapeutic index <90%; Poor Value value >90%.

S. No.	Phytochemical	Absorption (GI absorption)	Absorption P-glycoprotein substrate	Distribution (BBB permeability)	Distribution PPB	Metabolism (CYP2D6 inhibitor)	Excretion (OCT2 Substrate)	Toxicity (AMES)	Elimination criterion
1	Ugonin A	97.16	Inhibitor	−0.802	71.44	No	No	No	PAINS
2	Bismurrayaquinone A	100.0	Inhibitor	−0.29	53.1	No	No	No	PAINS
3	Argentine	97.15	Non-Inhibitor	−0.02	57.64	No	No	No	Hepatotoxic
4	Parvisoflavone B	88.31	Non-Inhibitor	−1.03	74.91	No	No	No	Hepatotoxic
5	Kuwanon D	100.0	Non-Inhibitor	−0.93	31.12	No	No	No	Poorly soluble
6	Bianthraquinone	100.0	Inhibitor	−0.10	98.29	No	No	No	PAINS
7	Californine	96.67	Non-Inhibitor	0.44	36.39	Yes	No	Yes	Toxicity
8	Withaphysalin D	97.53	Non-Inhibitor	−0.25	72.33	No	No	No	None
9	Nicandrenone	90.60	Inhibitor	0.10	53.77	No	No	No	None
10	Ovalichromene B	96.17	Inhibitor	0.16	66.28	No	No	No	Carcinogenicity
11	Phaseolin	95.01	Non-Inhibitor	0.14	80.34	No	No	No	None
12	Withaphysalin A	97.77	Non-Inhibitor	−0.26	71.65	No	No	No	Carcinogenicity
13	Picrasidine T	100.0	Non-Inhibitor	−1.42	74.79	No	No	No	Hepatotoxic
14	(−)-Ephedradine A	78.12	Non-Inhibitor	−0.36	75.74	No	No	Yes	Toxicity
15	Aphelandrine	78.12	Non-Inhibitor	−0.36	76.01	No	No	Yes	Toxicity

### 3.3 PASS analysis

PASS analysis suggests that compounds with a higher Pa (probability of being “Active”) than Pi (probability of being “Inactive”) are likely to show the desired biological activity. Pa represents the chance that the compound will have a specific biological effect, while Pi represents the chance that it will not show any particular activity (Filimonov et al., 2014). PASS evaluates chemical compound structures to predict diverse biological activities simultaneously. This computational tool is a valuable resource for estimating molecules’ potential biological activity profiles prior to their chemical synthesis and laboratory testing. Using the Way2Drug web-based platform, researchers conducted PASS analyses on the selected compounds to identify anti-cancer activities, aiming to uncover promising therapeutic candidates for cancers linked to KLK2 dysregulation (Druzhilovskiy et al., 2017). Low Pa values (≤0.3) in PASS predictions indicate a low probability of actual biological efficacy. In our study, the PASS analysis revealed that the screened compounds, Phaseolin, Withaphysalin D, and Nicandrenone, exhibited promising biological activities, including antineoplastic and apoptosis-agonist properties with Pa values ranging from 0.404 to 0.892. These findings suggest that the identified compounds might hold significant potential for anticancer applications through KLK2 inhibition. From an initial pool of 15 compounds, these three were selected for further evaluation based on multiple criteria, including compliance with RO5, absence of PAINS alerts, and their predicted biological activities. A summary of the PASS analysis results is provided in Table 3. The PASS analysis highlighted favorable biological activity profiles for Phaseolin, Withaphysalin D, and Nicandrenone, especially regarding apoptosis induction and antineoplastic effects.

**TABLE 3:** Biological activities of the screened phytochemicals predicted via PASS Server.

S. No.	Phytochemical	Pa value	Pi value	Biological activity
1	Phaseolin	0.822	0.007	Apoptosis agonist
		0.819	0.009	Antineoplastic
		0.404	0.021	Prostate cancer treatment
2	Withaphysalin D	0.892	0.005	Antineoplastic
		0.543	0.033	Apoptosis agonist
		0.499	0.014	Antineoplastic (lung cancer)
3	Nicandrenone	0.885	0.005	Antineoplastic
		0.557	0.014	Antineoplastic (breast cancer)
		0.430	0.060	Apoptosis agonist

### 3.4 Interaction analysis

**FIGURE 1:**
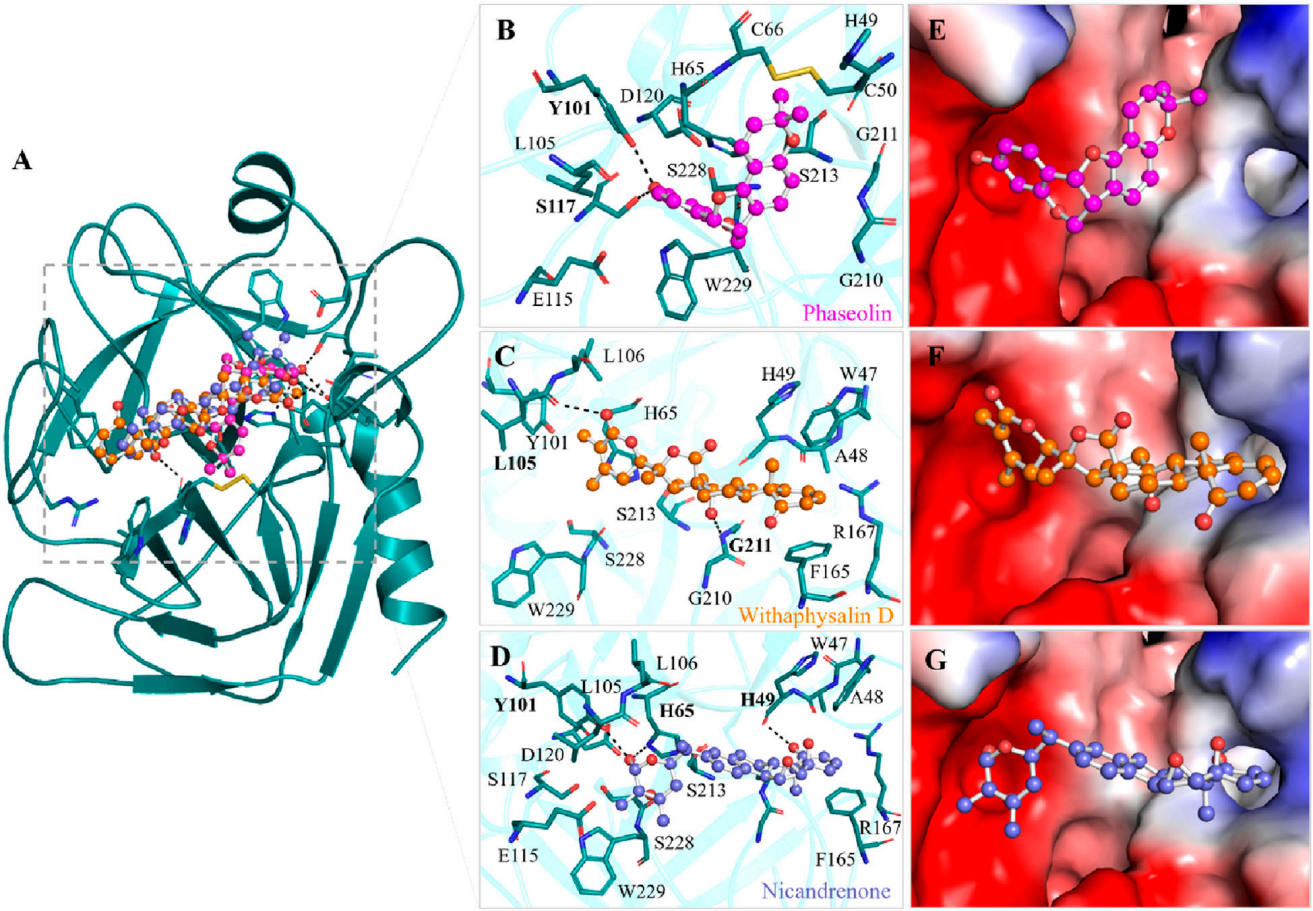
Binding prototype of Phaseolin (magenta), Withaphysalin D (orange), and Nicandrenone (blue) with KLK2. **(A)** Cartoon representation of KLK2 bound with the elucidated compounds. **(B)** Magnified view of KLK2 binding pocket interacting with Phaseolin, **(C)** Withaphysalin D, and **(D)** Nicandrenone. **(E)** Surface potential representation of the KLK2 binding pocket occupied by Phaseolin, **(F)** Withaphysalin D, and **(G)** Nicandrenone. Hydrogen bonds with a distance shorter than 3.5 Å are shown as black dotted lines and relevant residues are labelled with bold numbers. These representations were generated through PyMOL using the protein-ligand docked complexes from the docking study.

Interaction analysis is a pivotal technique in drug discovery, offering critical insights into the nature of bonds and residual interactions between a receptor and its ligand. In this study, the selected compounds, Phaseolin, Withaphysalin D, and Nicandrenone, exhibited strong interactions with key residues within the KLK2 binding site (Figure 1). Specifically, these compounds formed interactions with His65, Asp120, and Ser213, the active site residues essential for KLK2 activity (Figures 1B–D). A detailed depiction of their binding patterns is provided in Figure 1A, while the structural representations in Figures 1E–G highlight the compounds bound within the deep binding pocket of KLK2. The binding interactions observed suggest a significant potential for Phaseolin, Withaphysalin D, and Nicandrenone to inhibit KLK2 activity. The ability of these compounds to occupy the deep binding pocket and establish stabilizing interactions with His65, Asp120, and Ser213 is particularly notable, as these residues play a crucial role in KLK2’s enzymatic activity. Further studies should focus on refining the structural attributes of these compounds to enhance specificity and potency.

**FIGURE 2:**
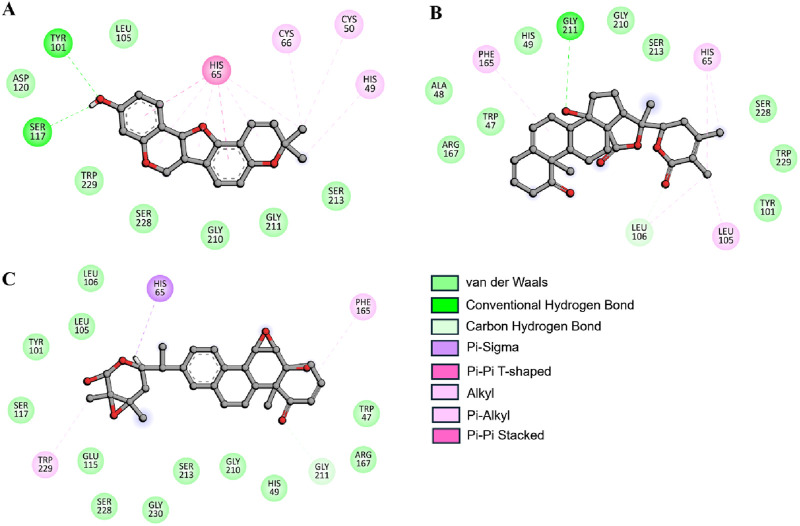
Depiction of molecular interactions and 2D plots illustrating detailed interactions for **(A)** Phaseolin, **(B)** Withaphysalin D, and **(C)** Nicandrenone.

All the three compounds demonstrated robust binding interactions with KLK2’s active site, particularly engaging critical residues such as His65, Asp120, and Ser213. These residues are integral to the enzymatic activity of the peptidase S1 domain, as they participate directly in the catalytic mechanism of the protease. The S1 domain itself plays a crucial role in enzymatic functions, particularly proteolysis, which governs substrate recognition and cleavage. Computational analyses (Figure 2) revealed consistent interaction patterns across all compounds with KLK2’s binding site. The analysis showed that Phaseolin, Withaphysalin D, and Nicandrenone formed stable binding conformations with KLK2, (Figures 2A–C). Notably, these ligands exhibited deep penetration and complementary shape matching within the binding pocket, suggesting high target specificity and inhibitory potential. Such precise and energetically favorable interactions underscore the compounds’ suitability as candidates for disrupting KLK2-mediated pathways, which may translate to therapeutic applications. KLK2 functions in a prostate-specific biochemical environment, where pH (7.2–8.2) and zinc concentrations (1–3 mM) are critical in its enzymatic activity (Oliveira et al., 2024). Since KLK2 is a zinc-dependent protease, variations in zinc levels may influence inhibitor binding by altering protein conformation (Goettig et al., 2010). Additionally, the slightly alkaline pH of seminal fluid could affect the protonation state of ligands, potentially modifying their binding affinities (Pakkala, 2012). Future *in vitro* and *in vivo* studies should incorporate these physiological factors better to evaluate the efficacy and selectivity of KLK2 inhibitors.

### 3.5 MD simulation analysis

MD simulations utilize detailed models of the physics governing interatomic interactions to predict the movements of individual atoms within a protein or other molecular systems over time (Karplus and McCammon, 2002). MD simulations were conducted following virtual screening to evaluate the structural dynamics and conformational stability of KLK2-ligand complexes. The top candidates identified from the IMPPAT 2.0 library were simulated under physiologically relevant solvent conditions. Starting with their docked conformations, each system underwent a 300-ns simulation to assess structural and energetic parameters. Various structural and dynamic parameters were analyzed to monitor protein-ligand behavior. These simulations provided insights into the temporal evolution of KLK2’s structure, both in its unbound state and during ligand interactions. The data revealed how ligand binding induced conformational shifts and influenced the protein’s thermodynamic stability, offering mechanistic explanations for the observed inhibitory effects.

#### 3.5.1 Structural deviation

**FIGURE 3:**
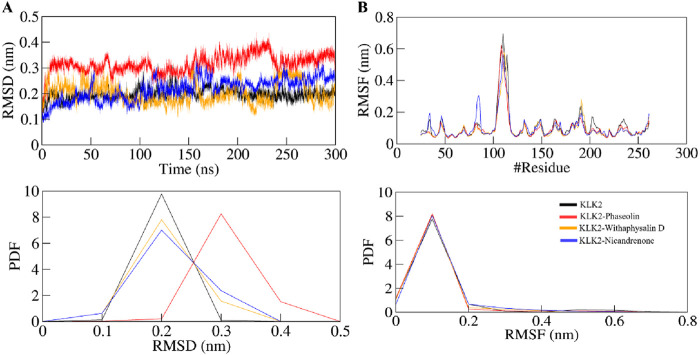
Structural dynamics of KLK2 upon binding with Phaseolin, Withaphysalin D, and Nicandrenone. **(A)** RMSD plot of KLK2 complex with Phaseolin, Withaphysalin D, and Nicandrenone. **(B)** RMSF plot of the KLK2 and its complex with Phaseolin, Withaphysalin D, and Nicandrenone. The lower panels depict the probability distribution function (PDF) of the values, with the position of the residues indicated by the symbol “#”.

In MD simulation, the root mean square deviation (RMSD) is a metric that quantifies the difference between two molecular structures, usually protein conformations. RMSD is frequently employed in drug discovery methods to monitor changes in molecular conformation over time in simulations and gain an understanding of the stability and dynamics of the system. A lower RMSD indicates more similarity, while a greater RMSD suggests significant structural variations. During MD simulation, we analyzed the RMSD pattern’s time evolution for KLK2 and its ligand-bound complexes with Phaseolin, Withaphysalin D, and Nicandrenone. The average RMSD values were calculated as 0.19 ± 0.02 nm, 0.31 ± 0.03 nm, 0.20 ± 0.04 nm, and 0.21 ± 0.04 nm for KLK2, KLK2-Phaseolin, KLK2-Withaphysalin D, and KLK2-Nicandrenone, respectively (Table 4). Figure 3A illustrates the structural behavior of KLK2 in complex with Phaseolin, Withaphysalin D, and Nicandrenone, revealing minor but consistent deviations. The KLK2-Withaphysalin D complex exhibited greater stability, as indicated by its lower RMSD values after binding. The RMSD plot in Figure 3A further supports this observation, showing fewer structural fluctuations in the KLK2-Withaphysalin D complex than the other two complexes. The relatively low RMSD values observed in KLK2-Withaphysalin D imply increased structural stability, a favorable property for maintaining inhibitory effects.

**TABLE 4:** The average values of different parameters determined following 300 ns simulations. All MD simulation values are reported with standard deviations calculated using block averaging (mean ± SD).

Protein/protein-ligand	RMSD (nm)	RMSF (nm)	*R*_g_ (nm)	SASA (nm^2^)	#H−Bonds
KLK2	0.19 ± 0.02	0.10 ± 0.10	1.71 ± 0.01	119.00 ± 2.84	144 ± 7
KLK2-Phaseolin	0.31 ± 0.03	0.09 ± 0.08	1.75 ± 0.01	121.21 ± 2.39	144 ± 7
KLK2-Withaphysalin D	0.20 ± 0.04	0.09 ± 0.08	1.71 ± 0.01	118.52 ± 2.28	144 ± 6
KLK2-Nicandrenone	0.21 ± 0.04	0.10 ± 0.07	1.71 ± 0.02	119.36 ± 3.07	142 ± 5

Root mean square fluctuation (RMSF) analysis was conducted to evaluate the flexibility and motion of individual residues in KLK2 before and after binding with the selected molecules. RMSF calculates the average fluctuation of residues, providing an understanding of their local flexibility and deviation from the mean positional coordinates. The distinctive protein backbone in all four systems, KLK2, KLK2-Phaseolin, KLK2-Withaphysalin D, and KLK2-Nicandrenone, showed RMSF values with noticeable peaks in the profiles. The average RMSF values observed were 0.10 ± 0.10 nm, 0.09 ± 0.08 nm, 0.09 ± 0.08 nm, and 0.10 ± 0.07 nm for KLK2, KLK2-Phaseolin, KLK2-Withaphysalin D, and KLK2-Nicandrenone, respectively. These results highlight the comparable stability and flexibility of the KLK2 protein across these interactions. Figure 3B presents the RMSF analysis, indicating that the KLK2-Withaphysalin D complex maintained greater stability with lower RMSF values compared to the other two complexes. However, all three complexes displayed a consistent and stable RMSF pattern throughout the simulation. The RMSF analysis revealed minor fluctuations in KLK2-ligand complexes, with lower flexibility at critical residues (His65, Asp120, and Ser213), suggesting that these compounds stabilize the active site effectively. Withaphysalin D showed particularly low RMSF values, indicating increased rigidity around the binding site, which is favorable for stable inhibition.

#### 3.5.2 Structural compactness

**FIGURE 4:**
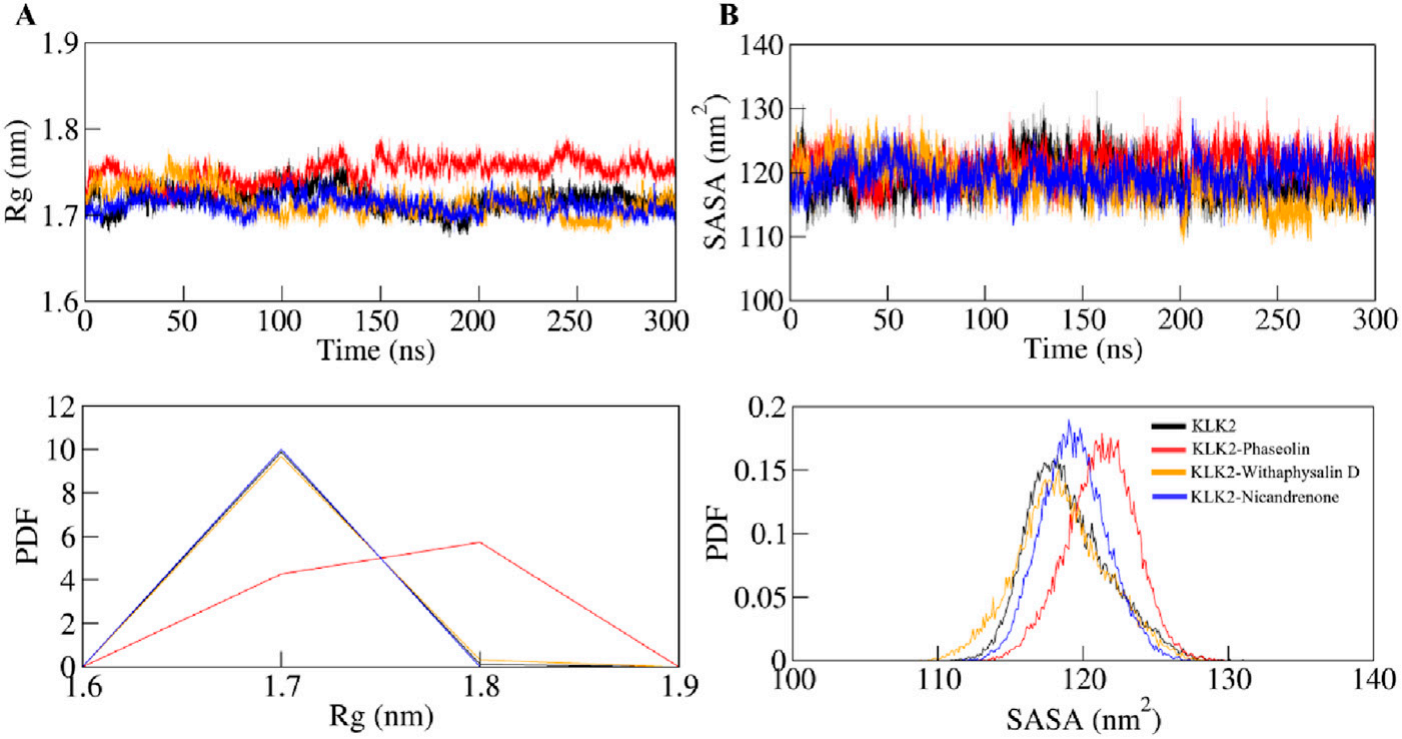
Time-evolution of the structural compactness and folding of KLK2 and its ligand-bound systems. **(A)** *R*_g_ plot and **(B)** SASA plot of KLK2 with Phaseolin, Withaphysalin D, and Nicandrenone. Lower panels show the probability distribution function as PDF.

The radius of gyration (*R*_g_) is a valuable parameter for evaluating the folding of a protein’s secondary structure into its tertiary structure, offering insights into its stability within a biological system. *R*_g_ measures the compactness of the protein by calculating the root mean square (RMS) distance of atoms from their collective center of mass. A reduction in the *R*_g_ value typically signifies a more compact and stable conformation during the formation of the protein-ligand complex (Lobanov et al., 2008). The average *R*_*g*_ values for KLK2, KLK2-Phaseolin, KLK2-Withaphysalin D, and KLK2-Nicandrenone were found to be 1.71 ± 0.01 nm, 1.75 ± 0.01 nm, 1.71 ± 0.01 nm and 1.71 ± 0.02 nm respectively. Figure 4A illustrates the time evolution of the *R*_*g*_, showing that all complexes maintained structural stability with consistent folding dynamics. Among them, the KLK2-Withaphysalin D complex exhibited greater compactness, indicated by its relatively lower *R*_*g*_ value, suggesting enhanced stability compared to the other complexes and the unbound state. The consistent *R*_g_ values across the simulation period suggest that binding does not induce significant unfolding, which is desirable for drug stability.

SASA is a critical parameter in molecular simulations that quantifies the extent of the protein surface accessible to solvent molecules. This measure reflects the interaction of the protein with its surrounding solvent, encompassing hydrophobic and hydrophilic residue (Dehdasht-Heidari et al., 2021). Figure 4B presents the SASA plot, where the average SASA values for free-state KLK2, KLK2-Phaseolin, KLK2-Withaphysalin D, and KLK2-Nicandrenone complexes were calculated. The analysis indicates that SASA remained unchanged throughout the simulation, suggesting consistent solvent exposure across all states. The average SASA values were calculated as 119 ± 2.84 nm^2^, 121 ± 2.39 nm^2^, 118 ± 2.28 nm^2^, and 119 ± 3.07 nm^2^ for KLK2, KLK2-Phaseolin, KLK2-Withaphysalin D, KLK2-Nicandrenone, respectively (Table 4). The SASA analysis demonstrated consistent values across all KLK2-ligand complexes, indicating that ligand binding does not compromise KLK2 compactness. Withaphysalin D showed the lowest SASA values, which, combined with its low RMSD and RMSF, further supports its suitability as a stable KLK2 inhibitor. Among the three compounds, Withaphysalin D showed the lowest RMSD and RMSF values, suggesting stronger and more stable binding to KLK2.

#### 3.5.3 Dynamics of hydrogen bonds

**FIGURE 5:**
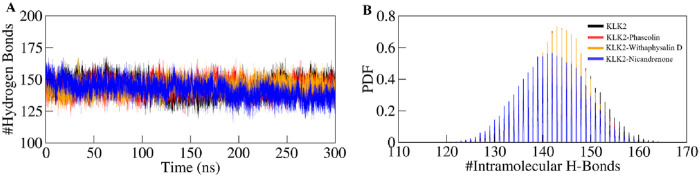
Hydrogen bond dynamics in KLK2. **(A)** Time evolution of intramolecular hydrogen bonds in KLK2 before and after the binding of Phaseolin, Withaphysalin D, and Nicandrenone. **(B)** The PDF of the hydrogen bond distribution in KLK2 systems. # represents a number.

Hydrogen bonds play a vital role in regulating the conformational dynamics of proteins (Kumar and Ojha, 2023). The intramolecular hydrogen bonding patterns were analyzed for unbound KLK2 structure and the KLK2 complexes bound to Phaseolin, Withaphysalin D, and Nicandrenone. The number of hydrogen bonds was monitored throughout the 300 ns simulation to assess the folding dynamics of KLK2 and its complexes (Figure 5A). The results revealed only slight variations in the number of intramolecular hydrogen bonds between the unbound protein and the three complexes. On average, the number of hydrogen bonds for the free KLK2 and its complexes with Phaseolin, Withaphysalin D, and Nicandrenone were 144 ± 7, 144 ± 7, 144 ± 6, and 142 ± 5, respectively (Table 4). The PDF of the intramolecular hydrogen bonds also demonstrated consistency and reliability across the systems (Figure 5B). The plots indicated that the hydrogen bonds in KLK2 remained stable throughout the simulation across all systems, with the complexes involving Phaseolin, Withaphysalin D, and Nicandrenone displaying consistent hydrogen bonding patterns comparable to the unbound KLK2. This stability underscores the structural integrity of KLK2 upon ligand binding and supports the notion that the binding of these compounds does not significantly disrupt the protein’s internal hydrogen-bonding network.

**FIGURE 6:**
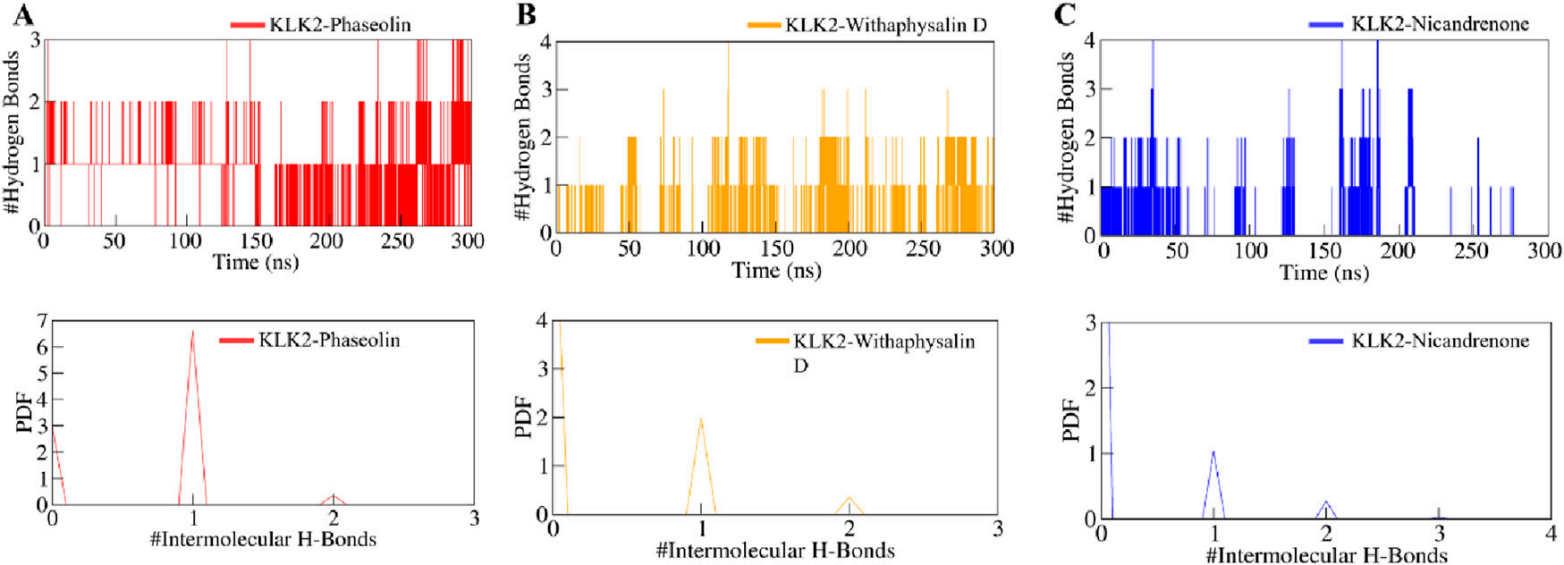
The dynamics of intermolecular hydrogen bonds formed between KLK2-ligand complexes. **(A)** Intermolecular hydrogen bonds between KLK2 and Phaseolin, **(B)** KLK2 and Withaphysalin D, and **(C)** KLK2 and Nicandrenone. The lower panel shows the probability distribution function plot.

Intermolecular hydrogen bonds formed between the selected compounds and KLK2 were analyzed to assess binding stability. All complexes exhibited stable hydrogen bonding interactions throughout the simulation (Figure 6). The KLK2-Phaseolin and KLK2-Withaphysalin D complexes had more hydrogen bonds than KLK2-Nicandrenone, and 2–4 hydrogen bonds persist throughout the 300 ns period in each complex (Figures 6A–C). The KLK2-Nicandrenone complex maintained 1–3 persistent hydrogen bonds throughout the simulation (Figure 6C). These results indicate minimal structural changes in the protein-ligand complexes over time. The stability of intermolecular hydrogen bonds contributed to preserving the initial docking conformation during the simulation. Stable hydrogen bonding was observed within the KLK2-ligand complexes, with KLK2-Withaphysalin D and KLK2-Phaseolin forming the most persistent hydrogen bonds. This suggests that these compounds establish strong, stable interactions with KLK2, critical for their efficacy as inhibitors.

#### 3.5.4 Evaluation of secondary structure

**FIGURE 7:**
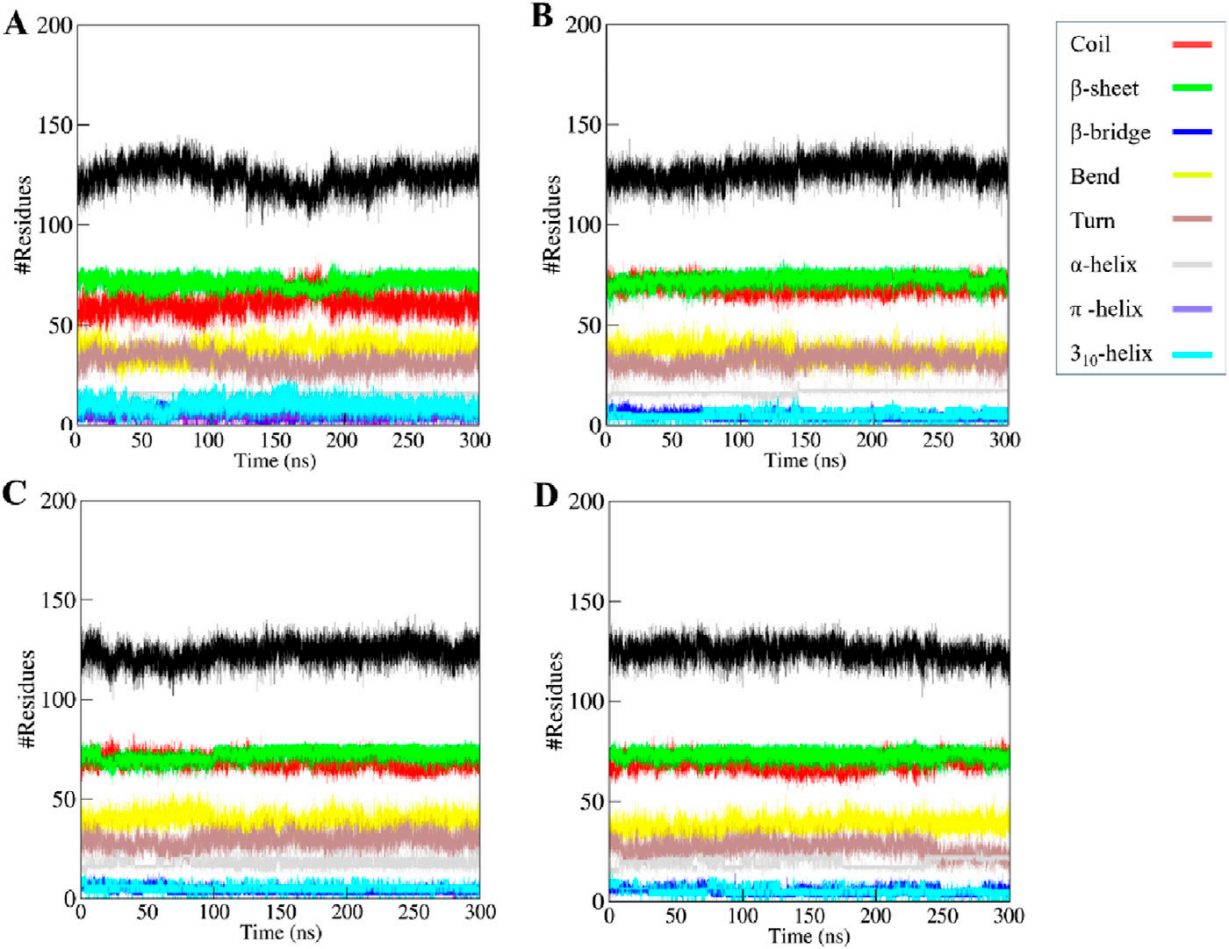
The secondary structure content of **(A)** KLK2 **(B)** KLK2-Phaseolin **(C)** KLK2-Withaphysalin D, and **(D)** KLK2-Nicandrenone.

Studying how a protein’s secondary structure changes can help us understand how it behaves and how it folds (Cino et al., 2012). We studied how the structure of KLK2 changes when it binds to three different compounds: Phaseolin, Withaphysalin D, and Nicandrenone. The structure of KLK2 stays mostly the same and stable during the 300 ns simulation (Figure 7A). However, there is a slight change in the α-helix and β-sheets of KLK2 when the compound binds (Table 5). The KLK2-Phaseolin complex exhibited slight variations in the average number of residues involved in secondary structure formation (Figure 7B), KLK2-Withaphysalin D (Figure 7C), and KLK2-Nicandrenone (Figure 7D) complexes as compared to the KLK2. No significant alterations were observed in the secondary structure of KLK2 upon binding with Phaseolin, Withaphysalin D, and Nicandrenone, indicating strong structural stability of the corresponding complexes. This preservation of the secondary structure highlights the compatibility of the selected compounds with KLK2, suggesting that their binding does not compromise the protein’s native structural integrity and may contribute to maintaining its functional conformation. Table 5 illustrates the number of residues involved in secondary structure elements of KLK2 before and after ligand binding. The analysis suggests that ligand binding had minimal impact on KLK2’s secondary structure, indicating a stable and non-disruptive interaction. This stability across α-helices and β-sheets supports the hypothesis that the identified compounds can inhibit KLK2 effectively without compromising its structural integrity.

**TABLE 5:** The average number of residues involved in KLK2’s secondary structure elements analyzed before and after ligand binding.

Element	KLK2	KLK2-Phaseolin	KLK2-Withaphysalin D	KLK2-Nicandrenone
Coil	61	69	68	68
β-sheet	71	73	72	73
β-bridge	5	4	4	5
Bend	37	36	39	38
Turn	32	32	29	27
α-helix	16	17	18	20
Π-helix	0	0	0	0
3_10_-helix	5	5	6	5
PPII-Helix	9	0	0	0

### 3.6 Principal component analysis

**FIGURE 8:**
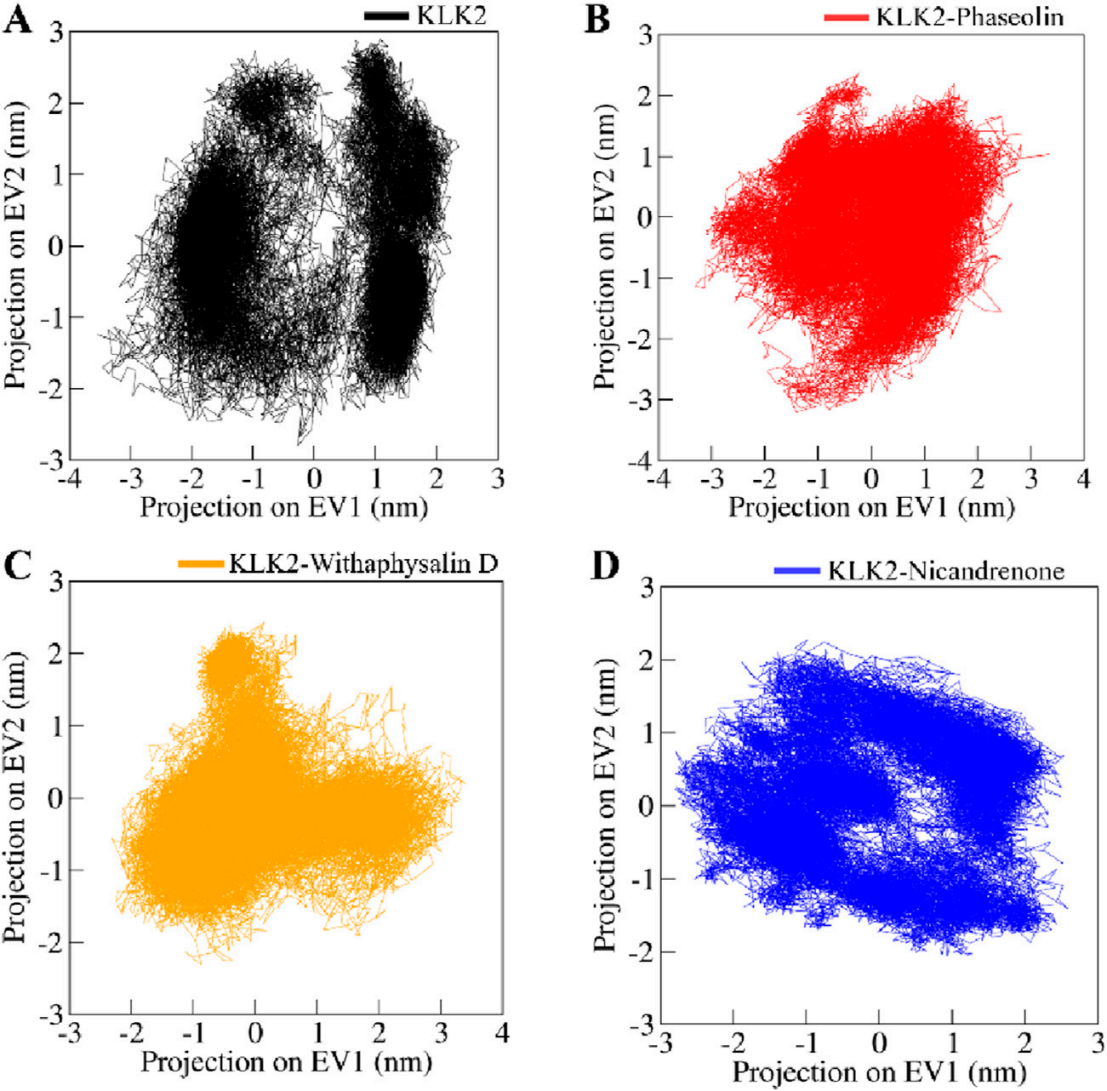
Conformational projections of **(A)** KLK2, **(B)** KLK2-Phaseolin, **(C)** KLK2-Withaphysalin D, **(D)** KLK2-Nicandrenone. The projection were generated through principal component analysis of the MD trajectories.

A widely used statistical approach in drug discovery, PCA, simplifies complex datasets by emphasizing the most significant variations. It is particularly effective in identifying conformational changes in biomolecules like proteins by transforming atomic motion data into principal components representing dominant motions. This approach helps analyze structural shifts and dynamic movements in protein-ligand complexes, offering valuable insights into their stability and interactions. This study utilized PCA to explore the conformational dynamics of KLK2 and its complexes with Phaseolin, Withaphysalin D, and Nicandrenone. Conformational sampling was performed by projecting the C^α^ atoms, as shown in Figure 8. Notably, the essential subspaces occupied by native KLK2 closely aligned with those of the protein-ligand complexes (Figures 8A–D), with none of the complexes exceeding the eigenvectors (EVs) observed in KLK2. The restricted conformational changes observed in PCA indicate that the ligand-bound KLK2 maintains its structural integrity, supporting the potential for stable and effective inhibition by these compounds.

### 3.7 Free energy landscape analysis

**FIGURE 9:**
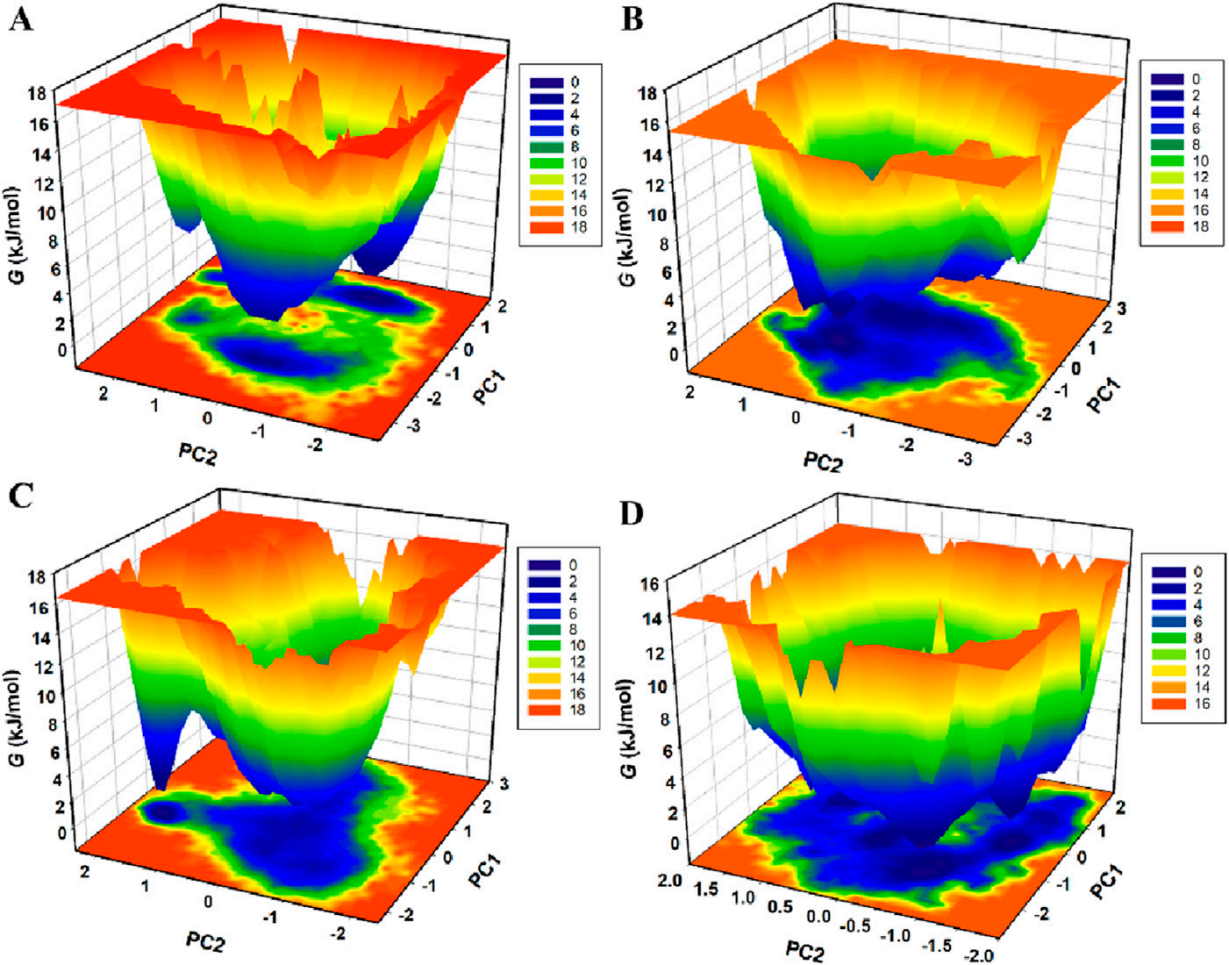
The Gibbs free energy landscapes of **(A)** KLK2 **(B)** KLK2-Phaseolin **(C)** KLK2-Withaphysalin D, and **(D)** KLK2-Nicandrenone.

FEL analysis was conducted to explore further the folding mechanisms and energetic landscape of the protein-ligand complexes under solvent conditions (Krivov, 2011). This method provides valuable information about the global and local energy minima that the complexes attain during simulations. Figure 9 illustrates the FELs of KLK2 in native form and the KLK2-Phaseolin, KLK2-Withaphysalin D, and KLK2-Nicandrenone complexes (Moritsugu et al., 2017). In FEL analysis, Gibbs free energy (*G*) is depicted through color gradients, where blue regions indicate low-energy, stable states and red regions represent high-energy, less favorable conformations. The native KLK2 structure exhibited fewer basins, corresponding to a global minimum (Figure 9A). Upon binding with Phaseolin and Nicandrenone, multiple basins appeared (Figures 9B–D), whereas Withaphysalin D binding resulted in a single, extensive basin (Figure 9C). These findings suggest that ligand binding subtly influenced the global minimum of KLK2. However, the FEL analysis confirmed that interactions with Phaseolin, Withaphysalin D, and Nicandrenone did not disrupt KLK2’s structural integrity, as the protein remained stable throughout the 300 ns simulation. The FEL analysis underscores the stability of KLK2 complexes with Phaseolin, Withaphysalin D, and Nicandrenone. The presence of distinct energy minima suggests these compounds stabilize KLK2 effectively, maintaining the low-energy conformations crucial for stable inhibition.

### 3.8 MM/PBSA analysis

MM/PBSA analysis was conducted to estimate the binding free energy of KLK2 protein-ligand complexes using the gmx_MMPBSA module in GROMACS. This method provides a thermodynamic measure of the energy change associated with ligand binding, offering insights into the strength and stability of protein-ligand interactions (Genheden and Ryde, 2015). Binding free energy components, including van der Waals forces and their corresponding standard deviations, were computed and summarized in Table 6. The results indicated that all KLK2-ligand complexes exhibited strong binding affinities, contributing to stable interactions. Among them, the KLK2-Phaseolin complex demonstrated the highest binding affinity (−14.34 kJ/mol), suggesting a particularly stable interaction. Conversely, the KLK2-Nicandrenone complex displayed the lowest binding free energy, indicating weaker binding stability. Overall, the analysis highlighted Phaseolin and Withaphysalin D as the most promising binders of KLK2, with superior binding affinities. These findings suggest that both compounds could be potential candidates for therapeutic development targeting KLK2.

**TABLE 6:** MM-PBSA calculations of binding free energy for KLK2-ligand complexes.

Complex	ΔVDWAALS	*ΔE*_*EL*_	Δ*E*_PB_	Δ*E*_NPOLAR_	Δ*G*_GAS_	Δ*G*_SOLV_	Standard deviation	${∆G}_{Total\;\left(kJ/mol\right)}$
KLK2-Phaseolin	−14.41	−18.40	20.46	−1.99	−32.81	18.47	2.38	−14.34
KLK2-Withaphysalin D	−16.39	−7.35	13.51	−1.96	−23.74	11.55	2.47	−12.19
KLK2-Nicandrenone	−0.68	−0.16	0.70	−0.11	−0.84	0.59	0.73	−0.25

Collectively, this study identifies Phaseolin, Withaphysalin D, and Nicandrenone as structurally distinct phytochemicals with robust KLK2 inhibitory potential, evidenced by their high binding affinities, stable molecular dynamics profiles, and complementary interactions with KLK2’s active site. Beyond its role in tumor progression, KLK2 is implicated in critical oncogenic processes, including epithelial-to-mesenchymal transition, angiogenesis, and extracellular matrix remodeling (Srinivasan et al., 2022). Inhibiting KLK2 may reduce metastatic potential by interfering with the proteolytic pathways required for cancer cell invasion and dissemination (Kryza et al., 2016). By attenuating KLK2’s proteolytic activity, these compounds may synergize with androgen deprivation therapies, counteracting resistance mechanisms in advanced prostate cancer. Recent advancements in ligand-directed delivery systems, such as those used for chemically modified miR-34a, offer promising strategies for enhancing the targeted delivery of KLK2 inhibitors (Abdelaal et al., 2024). These approaches could improve bioavailability, tumor selectivity, and therapeutic efficacy while minimizing systemic toxicity (Li et al., 2024). Integrating phytochemical-based KLK2 inhibitors with nanoparticle-based or ligand-mediated delivery systems could provide a more effective therapeutic strategy for prostate cancer treatment.

### 3.9 Limitations and future recommendations

This study, while comprehensive in its computational approach, has certain limitations. The absence of experimental validation remains a critical gap, as *in silico* predictions require empirical confirmation to establish therapeutic relevance. Additionally, the phytochemical library sourced from IMPPAT 2.0, though extensive, may not encompass all bioactive natural compounds, potentially overlooking candidates with unique binding mechanisms. To enhance structural diversity, future work should incorporate databases like ZINC Natural Products (https://zinc12.docking.org/), TCM Database (https://tcm.cmu.edu.tw/), and AfroDB (https://zinc12.docking.org/pbcs/afronp), which contain a broader range of bioactive compounds, including alkaloids and terpenoids. Another constraint is the reliance on static docking because dynamic protein ligand interactions in physiological conditions may be different (Olaoye et al., 2024). Future studies should, therefore, focus on the following recommendations: *In vitro* enzymatic assays using fluorogenic substrates to quantitate KLK2 inhibition and calculate IC_50_ values. Antiproliferative effects and apoptosis induction should be evaluated in cell-based assays in prostate cancer models; *in vivo* experiments could validate tumor suppression and pharmacokinetic profiles. Selectivity profiling against off-target proteases (e.g., KLK3, trypsin) is required for specificity, and co-crystallization studies of KLK2 ligand binding modes would provide structural insights for lead optimization. Finally, integrating ligand-directed delivery strategies, such as nanoparticle encapsulation inspired by recent advances in miR-34a therapeutics (Li et al., 2022), could enhance bioavailability and tumor targeting. These recommendations aim to bridge computational predictions with translational impact, advancing phytochemical leads toward preclinical development as targeted KLK2 inhibitors for prostate cancer.

## 4 Conclusion

KLK2 plays a crucial role in the progression of several cancer types, particularly prostate cancer, and represents a promising target for therapeutic intervention. Although some KLK2 inhibitors have been reported in the previous literatures, more potent and selective KLK2 inhibitors are still required. In order to fill this gap, we used a high-throughput virtual screening method in combination with MD simulations to identify new KLK2 inhibitors. From the phytochemical library of IMPPAT 2.0, we selected three lead compounds which are Phaseolin, Withaphysalin D, and Nicandrenone. These compounds showed good binding and proper orientation within the KLK2 binding site, especially with important active site residues. MM/PBSA analysis and MD simulations also supported the structural and dynamic stability of these complexes, and no drastic changes were observed in any of the four systems. The results presented in this work indicate that these compounds can be used to design effective KLK2 inhibitors. Future studies should include enzymatic inhibition assays (e.g., fluorogenic substrate cleavage assays) to validate the computational findings to determine IC50 values for Phaseolin, Withaphysalin D, and Nicandrenone against KLK2. Such efforts will help establish their efficacy and selectivity, paving the way for potential clinical development of natural compound-based KLK2 inhibitors.

## Data Availability

The original contributions presented in the study are included in the article/supplementary material, further inquiries can be directed to the corresponding authors.
